# Diagnostic Performance of Host and Viral DNA Methylation Analysis in the Identification of Anal Intraepithelial Neoplasia and Cancer: Systematic Review and Meta-Analysis

**DOI:** 10.3390/healthcare12191951

**Published:** 2024-09-30

**Authors:** Narcisa Muresu, Mariangela Valentina Puci, Giovanni Sotgiu, Illari Sechi, Andrea Cossu, Manuela Usai, Andrea Fausto Piana

**Affiliations:** 1 Medical Management, Hygiene, Epidemiology and Hospital Infection, University Hospital of Sassari, 07100 Sassari, Italy; 2 Clinical Epidemiology and Medical Statistics Unit, Department of Medicine, Surgery and Pharmacy, University of Sassari, 07100 Sassari, Italy; mvpuci@uniss.it (M.V.P.); gsotgiu@uniss.it (G.S.); 3 Department of Medicine, Surgery and Pharmacy, University of Sassari, 07100 Sassari, Italy; illasechi@uniss.it (I.S.); andreacossu@uniss.it (A.C.); piana@uniss.it (A.F.P.); 4 Department of Humanities and Social Science, University of Sassari, 07100 Sassari, Italy; manuelausai@hotmail.com

**Keywords:** HPV, anal cancer, DNA methylation test, biomarkers, epigenetic

## Abstract

Introduction: DNA methylation-based biomarkers have been investigated as useful tools in the carcinogenesis process, including the triage of HPV-associated cancers. In this context, we conducted a systematic review and meta-analysis focused on evaluating the changes in the level of DNA methylation in cases of pre-cancerous (i.e., anal intraepithelial neoplasia, AIN-1, -2., -3) and cancerous (i.e., squamous cell carcinoma, SCC) anal lesions. Methods: A research in the PubMed, Scopus, and Web of Science databases was carried out, following the PRISMA 2020 protocol, using the following keywords: “anal cancer”, “anal intraepithelial neoplasia”, “methylation”, and “epigenetic”. All observational studies that reported the level of DNA methylation by grade of anal lesions and for different target genes were included. The QUADAS-2 tool was used to assess the studies’ quality, whereas pooled prevalence, sensitivity, specificity, and diagnostic odds ratio (DOR) were employed to verify the accuracy of the test in the detection of high-grade lesions. Results: Eight studies met the inclusion criteria, involving a total of 1555 clinical samples. The prevalence of methylation-positive samples by histological grading was 27%, 45%, 54%, and 98% for AIN1, AIN2, AIN3, and SCC, respectively. Similar results were observed for the DOR, with higher ORs in more severe lesions. The pooled AUC (95%CI) for the diagnosis of ≥AIN2 was 0.68 (0.63–0.73). Conclusions: The present review and meta-analysis support the introduction of DNA methylation-based biomarkers in the triage of subjects with low-grade anal lesions and in the monitoring of treatment outcomes. Standardized protocols and a prospective study design are needed to implement methylation tests in clinical practice.

## 1. Introduction

The overall incidence and mortality of anal cancer in 2022 accounted for more than 54,000 and 21,000 cases, respectively, equally distributed in both sexes. Although anal cancer is a relatively rare disease in the general population, accounting for approximately 0.5% of all new cancer cases, a higher incidence has been evidenced in particular high-risk groups, such as people living with HIV and men who have sex with men (MSM) [1]. Moreover, a recent review evidenced a higher incidence in women than in men for the majority of countries, with the exception of Slovakia, Spain, Japan, and the Philippines. Anal squamous cell carcinoma (SCC) is the most common histopathological cancer, accounting for over 50% of diagnosed cases in both sexes, followed by adenocarcinomas, melanomas, sarcomas, and neuroendocrine tumors [2].

It has been estimated that >90% of anal SCC is related to Human Papillomavirus (HPV) infection; anal intercourse and having multiple sexual partners are known risk factors for infection and, consequently, contribute to an increased risk of anal cancer.

Since the identification of the etiologic role of HPV in anal cancer, based on cervical cancer nomenclature, a new classification was adopted: anal intraepithelial neoplasia (AIN), with AIN1, AIN2, and AIN3 grades, represents the precursor of anal cancer, whereas high/low-grade squamous intraepithelial lesions (HSILs/LSILs) are mainly related to HPV-16 infection [3]. Currently, recommendations do not include screening for HPV in the male population, besides offering an anal pap smear in subjects particularly at risk of developing anal pre-cancerous and cancerous lesions. Current guidelines of the International Anal Neoplasia Society (IANS) recommend screening interventions for people over 35 years old who are living with HIV, MSM, or transgender, as well as for men and women over the age of 45, without HIV. Moreover, the guidelines include screening interventions for solid organ transplant recipients (10 years after transplantation) and people with previous HPV-related diseases [2].

In recent years, emphasis has been placed on identifying viral markers that might have a role in the diagnosis and prognosis of HPV-related diseases, both in cervical and extra-cervical cancers [4,5]. Even if the natural history of HPV infection in anal dysplasia and cancer is still unclear, considering, unlike cervical cancer, the low rate of progression from AIN to cancer, several efforts have been aimed at identifying potential viral or human biomarkers able to predict the clinical course of anal pre-cancerous lesions, improving clinical outcomes and reducing overtreatment rates.

Recent advances in molecular and sequencing techniques have enabled a greater comprehension of epigenetic processes, such as histone modifications, DNA methylation, and non-coding RNA (nc-RNA), involved in the regulation of multiple cellular processes, including the progression/regression of carcinogenic lesions. In HPV-related cancer, both human and viral epigenetic targets are involved in carcinogenesis processes and potentially represent easily measurable tumor markers. In fact, it has been demonstrated that the methylation of DNA in cytosine–phosphate–guanine (CpG) sites through the addition of a methyl group to cytosine residues can activate or silence the function of that specific gene [6,7]. In particular, the mechanism behind DNA methylation in HPV-related cancers involves the hypermethylation of genes involved in regulatory regions of tumor suppression, resulting in progression to malignancy. To date, the most studied models are those concerning cervical lesions, for which it has been observed that DNA methylation is a reliable and valid triage tool for women who are HPV-positive in the prediction of disease outcomes [7].

Accordingly, the need to identify subjects at a high risk of progression becomes crucial in considering the most appropriate treatment and management of cases. However, differences in study design, endpoints, and methylation assays have limited the application of epigenetic tests for the diagnosis and prognosis of HPV-related cancer. On these bases, we conducted a systematic review and a meta-analysis aimed at evaluating the levels of DNA methylation in anal tissues with different histological grades and determine the accuracy of a methylation test in detecting high-grade pre-cancerous (i.e., AIN2 and AIN3) and cancerous lesions (i.e., SCC).

## 2. Materials and Methods

### 2.1. Study Design and Selection Criteria

A protocol for the present systematic review was developed following the PRISMA guidelines (Preferred Reporting Items for Systematic Review and Meta-analysis) [8], and this study was registered in the PROSPERO database (registration code: CRD42024559961) [9]. The PICO framework was used to define the eligibility criteria for the systematic review and meta-analysis, as follows: the patients (Ps) were all people screened for anal cancer and pre-cancerous lesions; intervention (I) was identified in all epigenetic analyses carried out via the identification of hypermethylated human and viral genes; the comparators (Cs) were represented by all cases without anal intraepithelial neoplasia (AIN) of grade 2 or worse (i.e., normal or AIN1 histological classification); and the outcome (O) was defined as the diagnostic accuracy of methylation testing in the diagnosis of pre-cancerous (i.e., ≥AIN2+) and cancerous (i.e., SCC) anal lesions.

All retrospective and longitudinal observational studies and cross-sectional studies published up until March 2024 were included in the present systematic review and meta-analysis, without any restrictions on the time and setting of the study.

A research was performed on the PubMed, Scopus, and Web of Science databases using MeSH terms such as “anal cancer”, “anal intraepithelial neoplasia”, “methylation”, “epigenetic”, and “HPV”, without any restriction in terms of time, setting, and demographic characteristics (e.g., age and/or gender). Moreover, all relevant research works reported in the reference list of each primary study were included in the review. After the removal of duplicates, titles and abstracts were independently screened by two authors (N.M. and I.S.), and full texts were assessed for eligibility. Any potential disagreements were resolved through the opinion of a third author (G.S.). All studies in the English language that were in accordance with the mentioned PICO model were included in the systematic review. Conversely, all research that did not define the histological and/or cytological characteristics of the samples tested and all studies that did not define the methodology of the methylation assay were excluded. Moreover, case reports or cases series with less than 10 subjects, as well as letters, editorials, and systematic reviews, were not considered.

### 2.2. Data Collection and Publication Bias

The data extracted included study characteristics (i.e., authors’ name, year of publication, country/countries of publication, and study design), population details (i.e., gender, age, and sample size), clinical characteristics (i.e., histological and/or cytological classification, HPV prevalence and genotyping, and HIV status), and intervention details (i.e., target gene, methodology of methylation assay, positivity of test by histological grading, and diagnostic accuracy of specific gene/s). An electronic form was developed to collect the studies’ variables, and it was implemented for qualitative and quantitative analysis, as well as for assessing the diagnostic accuracy of the methylation assay.

The grade of anal dysplasia was identified based on histological and/or cytological examinations following, respectively, the histologic terminology for HPV-associated lesions in the lower genital tract (intraepithelial neoplasia in the anal canal (AIN) divided into AIN1, AIN2, and AIN3) and the Bethesda System, which includes three main categories (i.e., low-grade squamous intraepithelial lesion (LSIL), high-grade squamous intraepithelial lesion (HSIL), and atypical squamous cells (ASCs) [10].

The risk of bias in each study was assessed according to the Quality Assessment of Diagnostic Accuracy Studies (QUADAS-2) criteria [11]. The quality of the studies was evaluated in terms of potential biases and applicability concerns through four domains related to patient selection, index tests, reference standards, and flow and timing of the intervention test.

### 2.3. Statistical Analysis

Descriptive statistics were used to summarize the main findings and demographic characteristics of the included studies. Meta-analytic estimates were calculated and reported with pooled and heterogeneity indicators. Forest plots were used to depict study variability, including 95% confidence intervals (CIs) for prevalence, sensitivity, and specificity measures, as follows: the size of the gray squares was proportional to the weight that each study contributed to the meta-analysis; the red diamond represented the overall estimated pooled measure; and the vertical dark line indicated the threshold for statistical significance. The effectiveness of the methylation test was measured as the diagnostic odds ratio, defined as the ratio of the odds of positivity in subjects with disease and positivity in subjects without diseases [12]. To assess the heterogeneity among the studies, the I^2^ statistic was calculated, with I^2^ values greater than 50% indicating substantial heterogeneity. Consequently, both fixed and random effect models were computed, accounting for the expected between-study heterogeneity. Funnel plots were generated to visually assess the publication bias based on Egger’s test. A two-tailed *p*-value < 0.05 was considered statistically significant. Data analyses were conducted using the STATA17 (StatsCorp, College Station, TX, USA), StatsDirect version 3.1.12 (StatsDirect Ltd., London, UK), and MetaDisc software (Meta-DiSc 2.0).

## 3. Results

**Figure 1:**
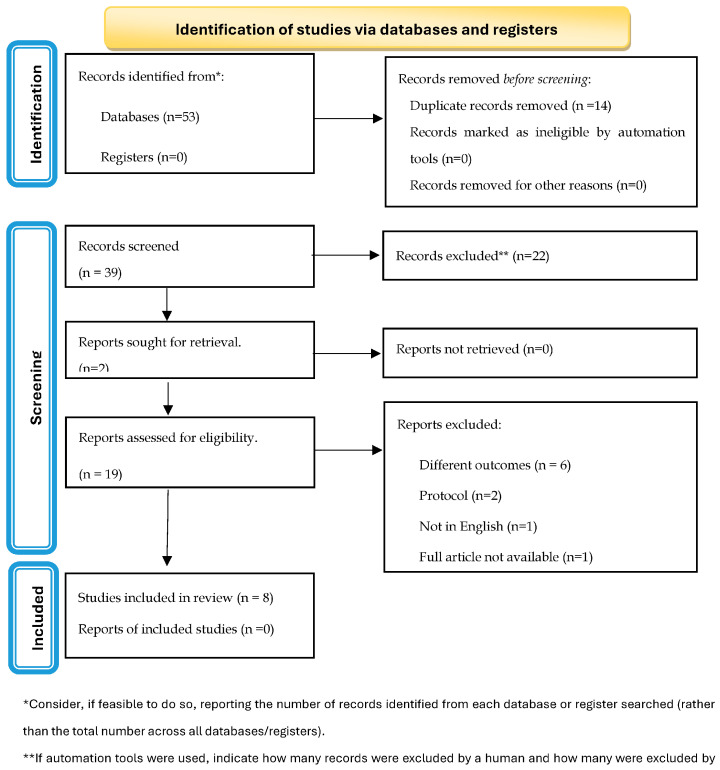
PRISMA flowchart of the search strategy results.

A total of 118 articles were retrieved from the Scopus, PubMed, and Web of Science databases, and 43.2% (51/118) were deleted as duplicates. One article was found in the reference list of another study, conducted by the same research team, and included in the review and meta-analysis [13]. After screening by title and abstract, 51 articles were removed as they did not meet the inclusion criteria (51/67; 76.1%). After full-text revision, eight studies (8/16; 50.0%) were excluded for the following reasons: five (31.3%) had missing information, one (6.3%) was a preliminary protocol, one (6.3%) was not in the English language, and one (6.3%) was not available. Of the 16 articles whose full text had been reviewed, 8 (50.0%) were included in the final analysis (Figure 1) [13,14,15,16,17,18,19,20].

### 3.1. Quality Assessment

Overall, all eight studies included in the systematic review showed a low risk of bias, with the only exception being the selection of patients, due to unclear enrolment methodologies [13,14,17,18,19,20]. Similarly, the quality assessment regarding applicability concerns demonstrated a low risk related to the index test and reference standard but remained high for patient selection [13,15,16,17,18,19,20] (Table 1) (Figure S1).

### 3.2. Study Characteristics

All studies were published between 2019 [13] and March 2024 [17], whereas the samples had been collected between 1985 [19] and 2020 [17,19]. Only one was a multicenter national study [17]. Most studies (5/8; 62.5%) had been carried out in Europe [13,17,18,19,20], whereas the remaining three had been conducted in South East Asia [14], the USA [15], and Australia [16]. Most of the studies were longitudinal or retrospective observational studies, except for two studies, which had been reported as having a cross-sectional design [15,17].

The sample population size ranged between 75 [15] and 429 participants [16], for a total of 1474 subjects involved. More than 88% of the patients were male (1189/1474; 80.7%), and only one study did not specify the sex of the patients involved. The age of the subjects ranged between 31 and 55 years [14,17], without significant differences between sexes. All studies reported the HIV status of the study population, with a range of positivity between 0% [19] and 100% [13,18,20], for a total of 1043 HIV-positive cases (Table S1).

### 3.3. Sample Description

All 1555 specimens analyzed were anal tissue samples, for the majority preserved as formalin-fixed paraffin-embedded tissue (873; 56.1%), while the rest were fresh biopsies (682; 43.9%) (Table S2).

The prevalence of HPV infections was reported in all studies and ranged between 37.5% [20] and 100% [14], for a total of 1249 HPV-positive cases (80.3%), one third of which (423; 33.8%) had been caused by HPV-16.

All studies reported patients’ histological classification based on the Bethesda system [13,14,17,18,19,20] or the cytological results [15,16]. The cytological analysis, available for 502 specimens (32.3%), identified 130 cases of high-grade lesions (HSILs) and 372 low-grade lesions (LSILs), in total [15,16].

Moreover, a histological examination was reported for 1489 samples, including the following: 520 samples (520/1489; 35.0%) with a negative/normal histology, followed by 226 AIN1 (15.2%), 243 AIN2 (16.3%), 200 AIN3 (13.4%), and 121 anal squamous cell carcinomas (8.1%). Based on epithelial cell abnormality, two studies reported 49 (3.3%) low-grade lesions and 130 (8.7%) high-grade lesions [15,16].

### 3.4. DNA Methylation Test

The majority of the studies used the Quantitative Multiplex Methylation-Specific PCR (QM-PCR) method to quantify DNA methylation in host target genes [13,15,16,17,18,19,20], whereas only one study [14] used a pyrosequencing technique in the viral genome. All studies analyzed multiple target genes (Table S3). Only one study targeted HPV-16 early (CpG 31,37,43,52, and 58) and late (CpG 7136, 7145, 5600, 5606, 5609, and 5615) regions [14], while two studies used the mir124-2 gene [15,16], and one study considered FAM19A4 [15] and CADM1 with MAL genes [16]. The most common gene was ZNF582, reported in five studies in combination with a panel of target genes [13,17,18,19,20] (Table S4). When the results of the DNA methylation analysis were reported for multiple targets, they were included in the meta-analysis.

According to the primary aim of each study, the results of the DNA methylation test were stratified by histological classification. The percentage of methylation-positive results in samples classified as having a normal histology ranged between 0% [14,20] and 34.8% [17]; the positivity for single or multiple target genes for the methylation test in the AIN1 group ranged between 8.1% [18] and 29.4% [17]. Samples classified as AIN2 showed positivity in the methylation test in 19–62% of cases [13,19]. Similarly, the positivity observed in AIN3 ranged between 29% and 73% [18,20]. Moreover, all samples classified as SCC showed a positivity higher than 93% [17] (Table 2).

Lastly, the positivity for a methylation target in the LSIL and HSIL specimens ranged between 57.5 and 45.8% and between 85 and 58%, respectively [15,16].

The sensitivity of the methylation test in ≥AIN2 detection ranged between 0.28 (95% CI: 0.15–0.44) [14] and 0.85 (95% CI: 0.55–0.98) [15], whereas the specificity was between 0.43 (95% CI: 0.27–0.60) [15] and 0.96 (95% CI: 0.87–1.0) [13].

### 3.5. Results of Meta-Analysis

**Figure 2:**
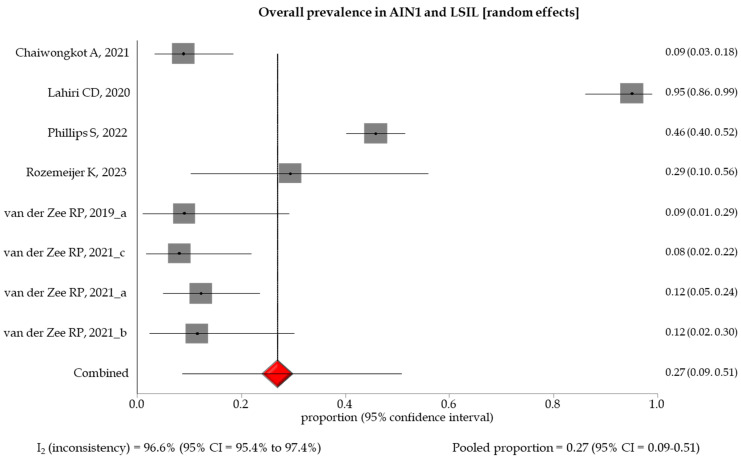
Forest plot of DNA methylation positivity in AIN1 and LSIL clinical specimens. The size of the gray squares is proportional to the weight that each study contributes to the meta-analysis. The red diamond represents the overall estimated pooled prevalence. The vertical dark line indicates the threshold for statistical significance (when the 95%CI includes the vertical line of “no effect”, the result is not statistically significant) [13,14,15,16,17,18,19,20].

**Figure 3:**
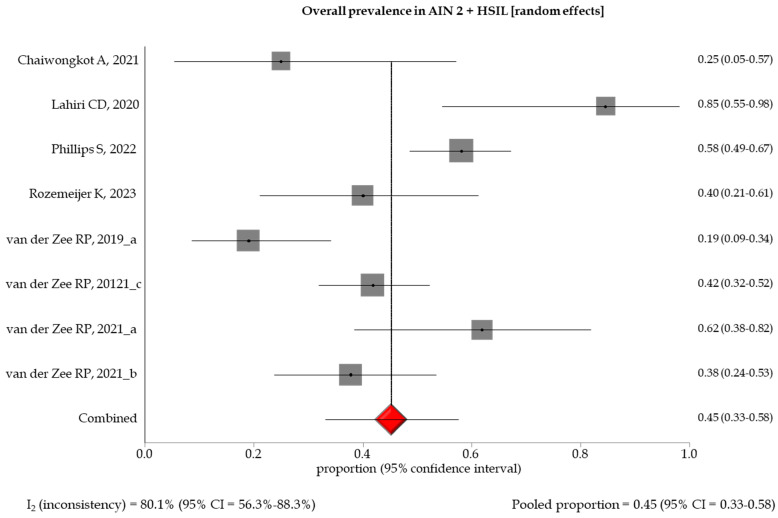
Forest plot of DNA methylation positivity in AIN2 and HSIL clinical specimens. The size of the gray squares is proportional to the weight that each study contributes to the meta-analysis. The red diamond represents the overall estimated pooled prevalence. The vertical dark line indicates the threshold for statistical significance (when the 95%CI includes the vertical line of “no effect”, the result is not statistically significant) [13,14,15,16,17,18,19,20].

**Figure 4:**
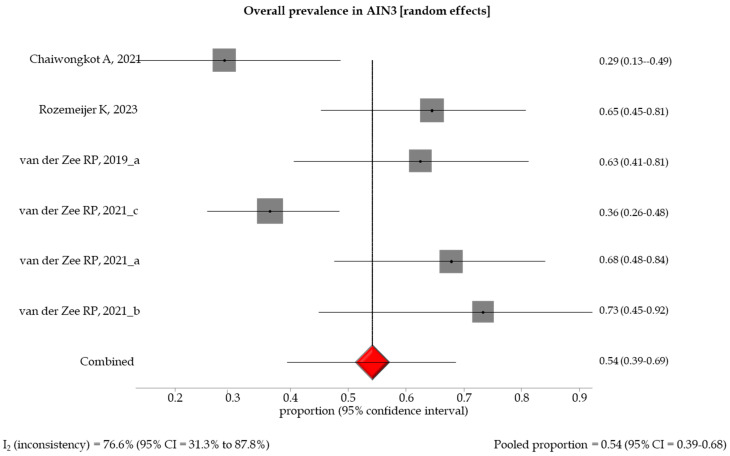
Forest plot of DNA methylation positivity in AIN3 clinical specimens. The size of the gray squares is proportional to the weight that each study contributes to the meta-analysis. The red diamond represents the overall estimated pooled prevalence. The vertical dark line indicates the threshold for statistical significance (when the 95%CI includes the vertical line of “no effect”, the result is not statistically significant) [13,14,17,18,19,20].

**Figure 5:**
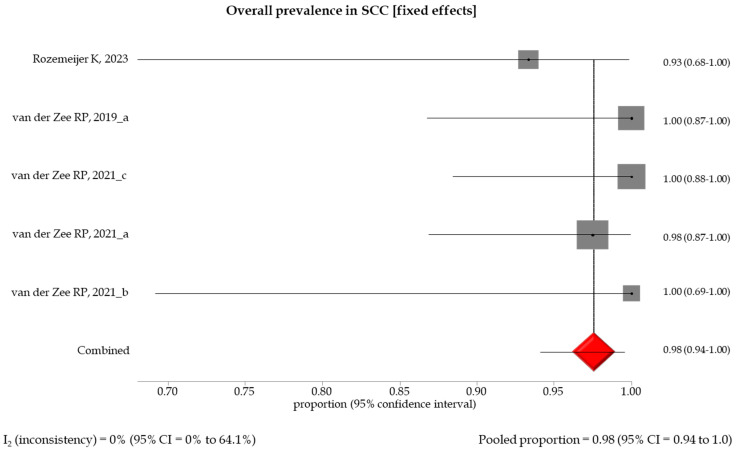
Forest plot of DNA methylation positivity in SCC clinical specimens. The size of the gray squares is proportional to the weight that each study contributes to the meta-analysis. The red diamond represents the overall estimated pooled prevalence. The vertical dark line indicates the threshold for statistical significance (when the 95%CI includes the vertical line of “no effect”, the result is not statistically significant) [13,17,18,19,20].

The pooled prevalence of methylation-positive results by different histological gradings showed an increasing trend between specimens with low- and high-grade anal lesions, with a pooled prevalence of 0.27 (95% CI: 0.09–0.51), 0.45 (95% CI: 0.33–0.58), 0.54 (95% CI: 0.39–0.68), and 0.98 (95% CI: 0.94–1.0) in AIN1 and LSIL, AIN2 and HSIL, AIN3, and SCC, respectively (Table 3, Figure 2, Figure 3, Figure 4 and Figure 5). A random effect model was applied in the stratified meta-analyses, considering the heterogeneities among the studies, except for the SCC group, in which a fixed model was adopted due to the non-existent heterogeneity (I_2_ = 0% (95% CI: 0.94–1.0). Egger’s test showed no significant evidence of publication bias in the studies (*p* > 0.5) (Figures S3–S6).

#### Diagnostic Accuracy of DNA Methylation Test in Detection of ≥ AIN2

**Figure 6:**
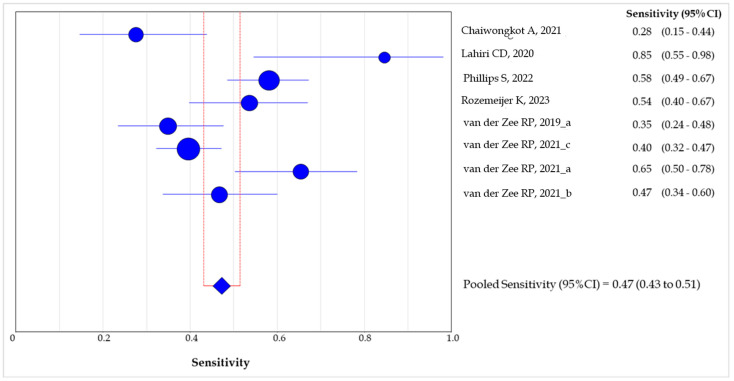
Forest plot of the pooled sensitivity of the methylation test in the detection of ≥2 AIN lesions among the included studies. The size of the blue dots is proportional to the weight that each study contributes to the meta-analysis. The blue diamond represents the overall estimated pooled sensitivity. The vertical red lines indicate the 95%CI for the pooled sensitivity [13,14,15,16,17,18,19,20].

**Figure 7:**
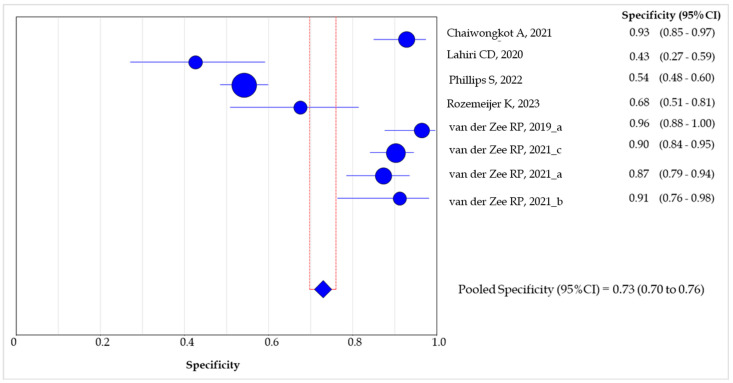
Forest plot of the pooled specificity of the methylation test in the detection of ≥2 AIN lesions among the included studies. The size of the blue dots is proportional to the weight that each study contributes to the meta-analysis. The blue diamond represents the overall estimated pooled specificity. The vertical red lines indicate the 95%CI for the pooled specificity [13,14,15,16,17,18,19,20].

The cumulative sensitivity of the DNA methylation test in the detection of lesions with grade ≥AIN2 was 0.47 (95% CI: 0.43–0.52) (Figure 6), whereas the pooled specificity was 0.73 (95% CI: 0.70–0.76) (Figure 7).

The diagnostic odds ratios for different histological gradings AIN1, AIN2, AIN3, and SCC were 1.1 (95% CI: 0.5–2.23) (*p*-value > 0.05), 3.1 (95% CI: 1.54–6.09) (*p*-value = 0.001), 7.46 (95% CI: 4.88–11.40) (*p*-value = 0.0001), and 162.37 (95% CI: 54.68–482.14) (*p*-value = 0.0001).

The pooled AUC of the methylation test for the diagnosis of ≥AIN2 grades was 0.68 (95%CI = 0.63–0.73) (Figure S2).

## 4. Discussion

The importance of considering secondary preventive strategies, such as screening, in populations at high risk of developing anal cancer has emerged only recently. These recommendations result from the well-known etiologic role of HPV infection in the occurrence of pre-cancerous lesions and the proven effectiveness of treatments in preventing them. Moreover, the IANS guidelines emphasize the need for individual screening protocols to implement in current tests (i.e., cytology and HPV tests). In this field, the increased methylation levels in host and viral genes serve as potential biomarkers for the early detection of high-grade anal intraepithelial neoplasia, offering a more tailored approach to screening [2].

The present systematic review and meta-analysis evaluated, for the first time in the current literature, how the level of DNA methylation changed in anal tissue specimens via histological grading, to consider its potential application as a diagnostic and prognostic biomarker in the detection of high-grade anal intraepithelial neoplasia and anal cancer. Despite the negligible heterogeneity among the studies, the results revealed a significant increase in DNA methylation positivity corresponding to the severity of histological grading. In detail, the pooled prevalence increased from 27% in negative histological samples to 54% in AIN3 and up to 98% in the SCC clinical samples. The high performance of this test is confirmed by the positivity found in almost all squamous cell tumors, with a percentage of positivity up to 98%. This is consistent with reports in the literature concerning HPV-associated cancers, for which an increase in the methylation level, both in viral and host cells, has been observed throughout the carcinogenesis process, offering a potential target for prevention and diagnosis [21,22].

In fact, considering the dynamic process of anal intraepithelial neoplasia, the identification of reliable and early markers for the diagnosis of pre-cancerous lesions seems particularly relevant in groups at a high-risk of developing cancer, such as people who are HIV-positive, MSM, and women with previous cervical lesions, distinguishing between those who require early treatment and those who need monitoring [1].

Our meta-analysis highlights the fact that the cumulative sensitivity and specificity of methylation testing showed good accuracy in detecting ≥AIN2 lesions. The cumulative sensitivity of the test was found to be 0.47, indicating that the test correctly identified 47% of high-grade lesions, but this highlights the need to cautiously interpret negative results. Conversely, the pooled specificity of 0.73 suggests that the test correctly identified 73% of non-high-grade lesions (i.e., grades below AIN2), with a moderate rate of false positives. These results could be partially influenced by the methodology and genomic target of the methylation assay. A recent review on the accuracy of methylation testing in the detection of pre-cancerous cervical lesions showed a high variability among the genes detected (i.e., mir124-2, FAM19A4, CADM1, MAL, etc.), evidencing the need for uniform protocols and cut-offs for different clinical specimens [23]. The high variability in the target genes of methylation assays and the use of multiple panels made it impossible to assess the exact accuracy for each protocol. However, an in-depth analysis of the most commonly used genes, such as ZNF582 and mir124-2, showed comparable results among the different studies and the good reliability and accuracy of the test. Previously, the frequent silencing of ZNF582 by methylation in cervical cancers was demonstrated, alongside its potential use for detecting cervical pre-cancerous lesions [24]. Similarly, miR124-2 methylation, usually in combination with FAM19A4, has emerged as a promising tool in women with positive HPV screening with a high negative predictive value for the progression of the lesions, limiting the overtreatment rate in young women [7,23]. It has been pointed out that the targets used are based on previous studies focusing on cervical specimens. Although there is a strong similarity in the pattern of HPV-related lesions, it would be important to identify specific genes for the different tissues and anatomical sites involved in HPV infections, ideally by genome-wide methylation profiling analyses [25].

The statistically significant difference in the diagnostic odds ratio among different histological grades confirmed the ability of the methylation assay to effectively discriminate patients with and without high-grade anal lesions and those with cancer diagnoses. Moreover, the strict confidence interval among the studies indicates the low variability and high performance of the test, independently of protocols and clinical settings. These findings suggest that the test is becoming progressively more effective in distinguishing between higher-grade lesions, particularly in the detection of squamous cell carcinoma.

The present review demonstrated a clear association between progression in anal lesions and the level of DNA methylation, supporting the applicability of this method in the monitoring and prevention of anal cancer during follow-ups, particularly in high-risk groups. The number of subjects involved and the low risk emerged in the quality assessment of the studies strengthen the reliability of this review and the meta-analysis results.

Despite offering valuable insights, this systematic review exhibited moderate heterogeneity in several aspects, such as setting design, protocols, population, and outcome reporting. This variability limited the generalizability of the findings and weakened their overall impact. Additionally, the small number of available studies contributed to limited data, while the quality assessment revealed a moderate risk of bias, primarily due to unclear criteria for patient enrollment. This ambiguity in the selection processes emphasizes the need for more rigorous research.

The key points that could be assessed for the applicability of methylation assays in clinical practice are related to the identification of the most affordable targets in host or viral cells, with defined cut-off values, and the implementation of long-term prospective studies aimed at defining the prognostic value and accuracy of this test within a screening protocol for the prevention of HPV-related diseases. Larger, well-designed prospective studies should focus on exploring the use of methylation markers in different populations and clinical settings to guide early detection and personalized treatment strategies.

## 5. Conclusions

Methylation tests represent a significant advancement in the diagnosis and management of HPV-related cancers. They offer potential for the earlier and more accurate detection of pre-cancerous lesions and cancers, particularly when used in combination with standard techniques, such as histological and cytological examinations and genotype-specific HPV testing. However, the adoption of this method in current diagnostic protocols is limited, mainly due to the current lack of standardized protocols, urgently needed to ensure the consistency and accuracy of the test and reduced the cost and time for the analysis. Overcoming these limitations will be useful for implementing epigenetic analyses within routine screening protocols and as biomarkers in treatment monitoring. Furthermore, the level of DNA methylation could be assessed during personalized diagnoses through specific genomic panels for different HPV-related cancers, using both tissue and liquid biopsies.

## Figures and Tables

**Table 1:** Bias and applicability evaluation of the included studies with the Quality Assessment of Diagnostic Accuracy Studies (QUADAS-2) tool [11].

Risk of Bias					Applicability Concerns		
	Patient Selection	Index Test	Reference Standard	Flow and Timing	Patient Selection	Index Test	Reference Standard
Chaiwongkot A, 2021 [14]	Low Risk	Low Risk	Low Risk	Low Risk	Low Risk	Low Risk	Low Risk
Lahiri CD, 2020 [15]	Low Risk	Low Risk	Low Risk	Low Risk	High Risk	Low Risk	Low Risk
Phillips S, 2022 [16]	High Risk	High Risk	Low Risk	Low Risk	High Risk	Low Risk	Low Risk
Rozemeijer K, 2023 [17]	High Risk	High Risk	Low Risk	Low Risk	High Risk	Low Risk	Low Risk
van der Zee RP, 2019_a [13]	High Risk	Unclear Risk	Low Risk	Unclear Risk	High Risk	Low Risk	Low Risk
van der Zee RP, 2021_c [18]	High Risk	Unclear Risk	Low Risk	Low Risk	High Risk	Unclear Risk	Low Risk
van der Zee RP, 2021_a [19]	High Risk	Unclear Risk	Unclear Risk	Low Risk	High Risk	Low Risk	Low Risk
van der Zee RP, 2021_b [20]	High Risk	Unclear Risk	Low Risk	Low Risk	High Risk	Unclear Risk	Low Risk

**Table 2:** Results of DNA methylation by histological classification of anal tissue samples.

N	First Author	Target Genes Considered for SR	Methylation Positive by Histological Classification, *n* (%)						
			Normal Histology	AIN 1	AIN 2	AIN 3	SCC	HSIL	LSIL
[14]	Chaiwongkot A, 2021	L1 gene (CpG 5600)	0/16 (0.0)	6/67 (10.5)	3/12 (25)	8/28 (28.6)			
[15]	Lahiri CD, 2020	FAM19A4/miR124–2						11/13 (84.6)	23/40 (57.5)
[16]	Phillips S, 2022 §	miR124–2						68/117 (58.1)	143/312 (45.8)
[17]	Rozemeijer K, 2023	Panel	8/23 (34.8)	5/17 (29.4)	10/25 (40)	20/31 (64.5)	14/15 (93.3)		
[13]	van der Zee RP, 2019_a	Panel	1/34 (2.9)	2/22 (9.1)	8/42 (19.0)	15/24 (62.5)	26/26 (100.0)		
[18]	van der Zee RP, 2021_c	Panel	11/106 (10.4)	3/37 (8.1)	41/98 (41.8)	27/74 (36.5)	30/30 (100.0)		
[19]	van der Zee RP, 2021_a	Panel	4/30 (13.3)	7/57 (12.3)	13/21 (61.9)	19/28 (67.9)	39/40 (97.5)		
[20]	van der Zee RP, 2021_b	Panel	0/8	3/23 (11.5)	17/45 (37.8)	11/15 (73.3)	10/10 (100)		

§ LSIL + Normal = Neg.

**Table 3:** Comparison and diagnostic accuracy of methylation analysis in the detection of <AIN2 vs. ≥AIN2 anal lesions.

N	Study ID	Target Gene/s	<AIN2 Histology, *n*		≥AIN2 Histology, *n*		Sensitivity (95% IC)	Specificity (95% IC)
			Methylation POS	Methylation NEG	Methylation POS	Methylation NEG		
[14]	Chaiwongkot A, 2021	L1 gene (CpG 5600)	6	77	11	29	0.28 (0.15–0.44)	0.93 (0.85–0.97)
[15]	Lahiri CD, 2020	FAM19A4/miR124–2	23	17	11	2	0.85 (0.55–0.98)	0.43 (0.27–0.60)
[16]	Phillips S, 2022	miR124–2	143	169	68	49	0.58 (0.49–0.67)	0.54 (0.49–0.60)
[17]	Rozemeijer K, 2023	Panel	13	27	30	26	0.54 (0.40–0.67)	0.68 (0.51–0.81)
[13]	van der Zee RP, 2019_a	Panel	2	54	23	43	0.35 (0.24–0.48)	0.96 (0.87–1.0)
[18]	van der Zee RP, 2021_c	Panel	14	129	68	104	0.40 (0.32–0.47)	0.90 (0.84–0.95)
[19]	van der Zee RP, 2021_a	Panel	11	76	32	17	0.65 (0.50–0.78)	0.87 (0.79–0.94)
[20]	van der Zee RP, 2021_b	Panel	3	31	28	32	0.47 (0.34–0.60)	0.91 (0.76–0.98)

Pooled sensitivity (95% IC): 0.47 (0.43–0.52); pooled specificity (95% IC): 0.73 (0.70–0.76).

## Data Availability

The data are available upon motivated requests.

## Supplementary Materials

The following supporting information can be downloaded at https://www.mdpi.com/article/10.3390/healthcare12191951/s1, Figure S1: QUADAS-2 tool; Table S1: Characteristics of the study and demographic data; Table S2: HPV positivity, histological classification of samples included in the systematic review; Table S3: Target genes of DNA methylation test in the included studies; Table S4: Results of DNA methylation by single target gene; Figure S2: ROC curves; Figures S3–S6: Funnel plots.
