# Supplementary material for: Diagnostic Performance of Host and Viral DNA Methylation Analysis in the Identification of Anal Intraepithelial Neoplasia and Cancer: Systematic Review and Meta-Analysis
Source: Healthcare (Basel). 2024 Sep 30;12(19):1951. doi: 10.3390/healthcare12191951 (PMC11475795; doi:10.3390/healthcare12191951)
Supplement: Supplementary file 1 [file healthcare-12-01951-s001.zip › healthcare-3181289-supplementary.pdf]

Figure S1_Supplementary: Bias and applicability evaluation of included studies with Quality Assessment of Diagnostic Accuracy Studies (QUADAS-2) tool.*

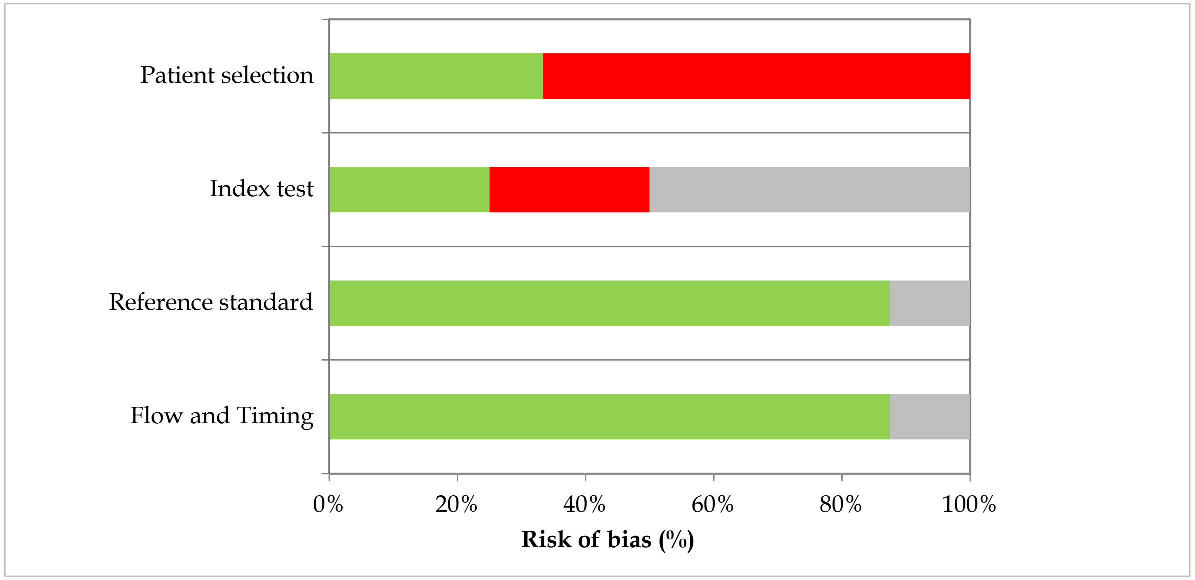

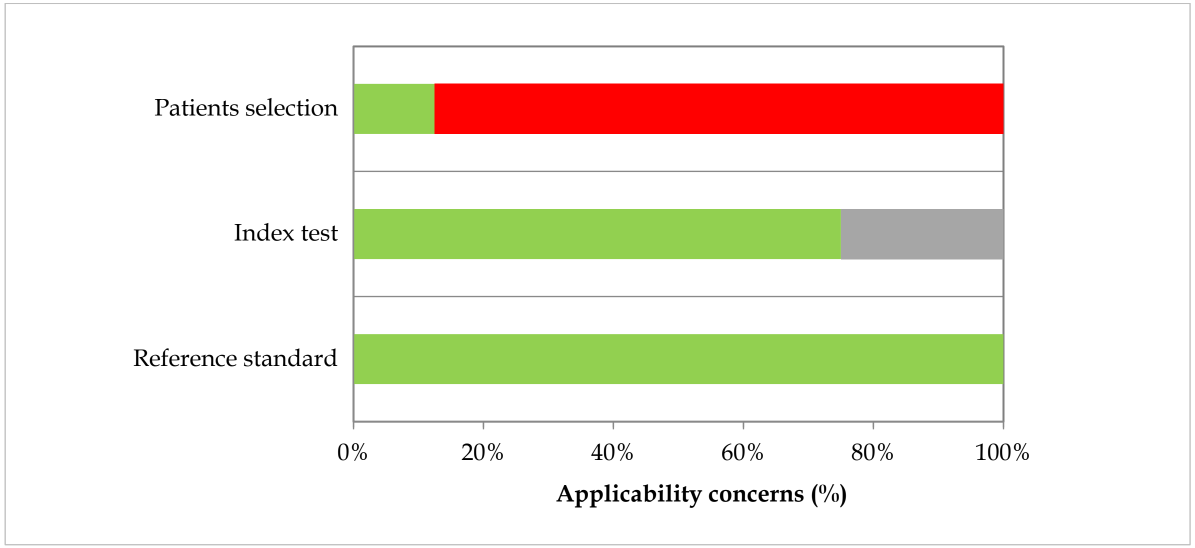

* Green: Low risk; Grey: Unclear risk; Red: High risk

Table S1_Supplementary. Characteristics of the study and demographic data.

n	First Author	Year	Study design	Mono /Multicentre	Country/ies	Samples collection (Y)	Sample size	Samples analysed	Age, mean/median (SD/IQR)*	Female, n (%)	Male, n (%)
1	*Chaiwongkot A; 2021*	2021	Observational-retrospective	Monocentre	Thailand	2013	178	178	31.2		
	Lahiri CD; 2020	2020	Cross-sectional	Monocentre	Atlanta		75	75	48.7 (8.5)	75 (100)	
3	*Phillips S; 2022*	2022	Observational	Monocentre	Australia		429	429	49 (35-75)		429 (100)
4	*Rozemeijer K; 2023*	2023	Cross-sectional	Multicentre	Netherlands	2018-2020	111	111	54.9 (10.4)	1 (0.1)	110 (99.1)
5	*van der Zee RP; 2019_a*	2019	Observational	Monocentre	Netherlands	1986-2011	122	122	48		148 (100)
6	*van der Zee RP; 2021_c*	2019	Cross-sectional + longitudinal	Monocentre	Netherlands	1999-2018	345	345	48 (47-53)		345 (100)
7	*van der Zee RP; 2021_a*	2021	Observational	Monocentre	Netherlands	1985-2020	121	176	52 (41-62)	57 (47.1)	64 (52.9)
8	*van der Zee RP; 2021_b*	2021	Observational	Monocentre	Netherlands	1999-2016	93	104			93 (100)

*SD/IQR: Standard Deviation/Interquartile range

Table S2_Supplementary. HPV positivity, histological classification of samples included in the systematic review.

n	First Author	Type of samples	HPV+, n (%)	Histological results, Tot. (n)	Normal Histology, n (%)	AIN 1, n (%)	AIN 2, n (%)	AIN 3, n (%)	SCC, n (%)	Low Grade AIN, n (%)	High Grade AIN, n (%)
1	*Chaiwongkot A; 2021*	Anal tissue	178 (100.0)	123	16 (13.0)	67 (54.5)	12 (9.8)	28 (22.8)			
2	*Lahiri CD; 2020*	Anal tissue	57 (77.0)	53	17 (32.1)					23 (43.4)	13 (24.5)
3	*Phillips S; 2022*	Anal tissue	315 (73.4)	429	286 (66.7)					26 (6.1)	117 (27.3)
4	*Rozemeijer K; 2023*	Anal tissue	68 (61.8)	111	23 (20.7)	17 (15.3)	25 (22.5)	31 (27.9)	15 (13.5)		
5	*van der Zee RP; 2019_a*	Anal tissue	139 (93.9)	148	34 (23.0)	22 (14.9)	42 (28.4)	24 (16.2)	26 (17.6)		
6	*van der Zee RP; 2021_c*	Anal tissue	305 (88.4)	345	106 (30.7)	37 (10.7)	98 (28.4)	74 (21.4)	30 (8.7)		
7	*van der Zee RP; 2021_a*	Anal tissue	148 (84.1)	176	30 (17.0)	57 (32.4)	21 (11.9)	28 (15.9)	40 (22.7)		
8	*van der Zee RP; 2021_b*	Anal tissue	39 (37.5)	104	8 (7.7)	26 (25.0)	45 (43.3)	15 (14.4)	10 (9.6)		

AIN: Anal Intraepithelial Neoplasia

Table S3_Supplementary. Target genes of DNA methylation test in the included studies.

n	First Author	Methylation test	Target genome methylation test	Target genes included
1	Chaiwongkot A; 2021	Pyrosequencing	Viral	HPV-16 early promoter (CpG 31,37,43,52, and 58);<br>L1 region (CpG 7136, 7145, 5600, 5606, 5609, and 5615)
2	Lahiri CD; 2020	Quantitative methylation-specific PCR	Host	FAM19A4, miR124-2
3	Phillips S; 2022 §	Quantitative methylation-specific PCR	Host	CADM1, MAL, miR124-2
4	Rozemeijer K; 2023	Quantitative methylation-specific PCR	Host	ASCL1, ZNF582, SST
5	van der Zee RP; 2019_a	Quantitative methylation-specific PCR	Host	ASCL1, GHSR, SST, ST6GALNAC3, ST6GALNAC5, WDR17, ZIC1, ZNF582, ZNF583
6	van der Zee RP; 2021_c	Quantitative methylation-specific PCR	Host	ASCL1, SST, ZNF582
7	van der Zee RP; 2021_a	Quantitative methylation-specific PCR	Host	ASCL1, ST6GALNAC3, WDR17, ZIC1, ZNF582
8	van der Zee RP; 2021_b	Quantitative methylation-specific PCR	Host	ASCL1, LHX8, ZNF582, SST, WDR17, ZIC1

§ LSIL+Normal=Neg

Table S4_Supplementary: Results of DNA methylation by single target gene.

**Study ID.**	**Hystological classification**	**ASCL 1**	**LHX8**	**SST**	**WDR17**	**ZIC1**	**ZNF582**	**miR124-2**	**MAL**	**CADM1**	**L1 gene (CpG 5600)**	**L1 gene (CpG 5609)**
Chaiwongkot A; 2021	Normal										0/16 (0.0)	0/16 (0.0)
	AIN1										6/67 (10.5)	2/67 (3.0)
	AIN2										3/12 (25.0)	2/12 (16.7)
	AIN3										8/28 (28.6)	2/28 (7.1)
Lahiri CD; 2020 *	HSIL							11/13 (84.6)				
	LSIL							23/40 (57.5)				
Phillips S; 2022	HSIL							68/117 (58.1)	76/117 (65.0)	54/117 (46.2)		
	LSIL							143/312 (45.8)	147/312 (41.1)	73/312 (23.4)		
Rozemeijer K; 2023 **	Normal						8/23 (34.8)					
	AIN1						4/17 (23.5)					
	AIN2						10/25 (40)					
	AIN3						20/31 (64.5)					
	SCC						14/15 (93.3)					
van der Zee RP; 2019_a	AIN1						13/22 (13.6)					
	AIN2						18/42 (42.9)					
	AIN3						13/24 (54.2)					
van der Zee RP; 2021_c	Normal						5/106 (4.7)					
	AIN1						1/37 (2.7)					
	AIN2						19/98 (19.4)					

	AIN3						30/30 (100.0)					
van der Zee RP; 2021_a	Normal	3/30 (10)	2/30 (7)	9/30 (30)	3/30 (10)	5/30 (17)	2/30 (7)					
	AIN1	10/57 (18)	7/57 (13)	6/57 (11)	5/57 (9)	4/57 (7)	5/57 (9)					
	AIN2	10/57 (18)	7/57 (13)	6/57 (1)	5/57 (9)	4/57 (7)	5/57 (9)					
	AIN3	18/28 (64)	18/28 (64)	22/28 (79)	18/28 (64)	19/28 (68)	18/28 (64)					
	SCC	37/40 (93)	33/40 (83)	39/40 (98)	38/40 (95)	37/40 (93)	37/40 (93)					
van der Zee RP; 2021_b	Normal						0/8 (0.0)					
	AIN1						3/23 (11.5)					
	AIN2						17/45 (37.8)					
	AIN3						11/15 (73.3)					
	SCC						10/10 (100)					

*FAM/miR124-2 combined
**ZNF582, ASCL1, SST combined

Figure S2_Supplementary: Estimated ROC curves with upper and lower limits of the 95% confidence intervals of methylation test in AIN2 diagnosis.

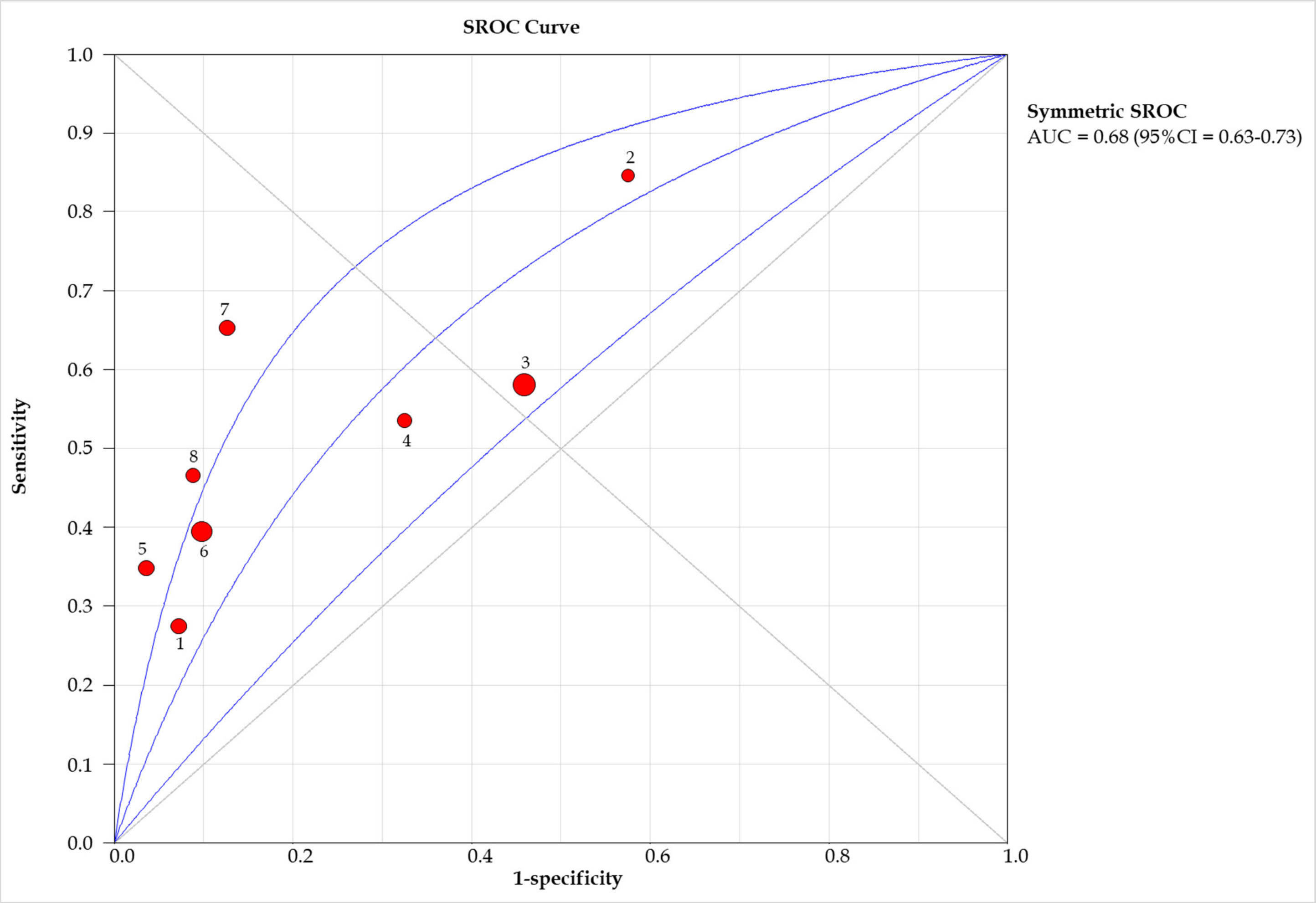

Figure 2: (1) Chaiwongkot A; 2021. (2) Lahiri CD; 2020. (3) Phillips S; 2022. (4) Rozemeijer K; 2023. (5) van der Zee RP; 2019_a. (6) van der Zee RP; 2021_c. (7) van der Zee RP; 2021_a. (8) van der Zee RP; 2021_b.

Figure S3: Funnel Plot for pooled proportion in AIN1 + LSIL

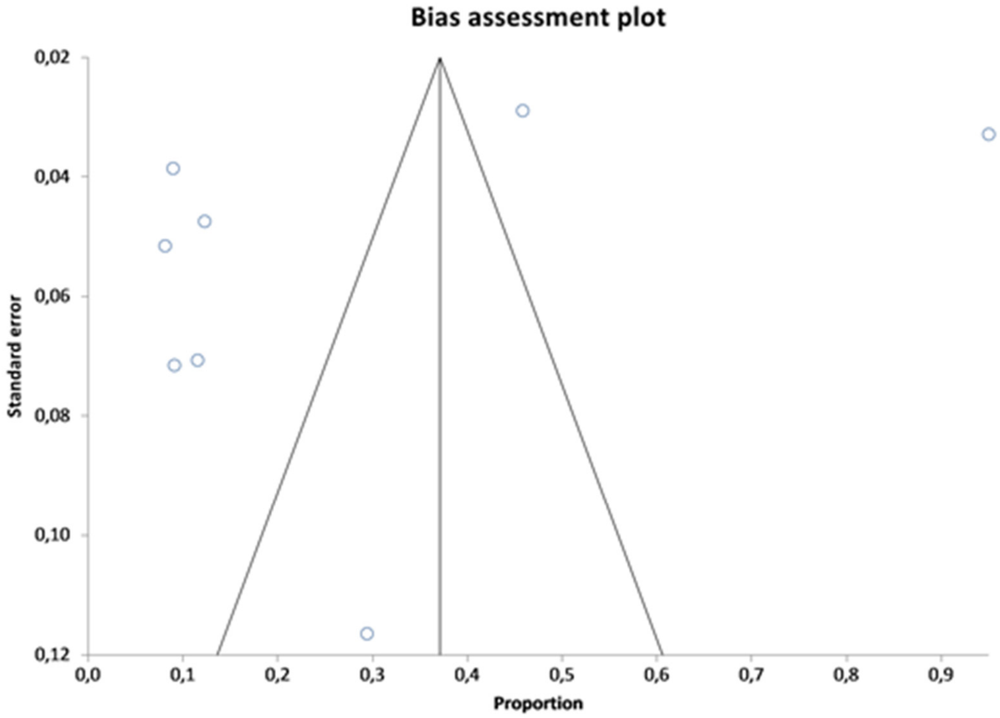

<u>Bias indicators:</u>

Egger: bias = -9,9 (95% CI = -27,9 to 8,1), P = 0,23

Figure S4: Funnel Plot for pooled proportion in AIN2 + HSIL

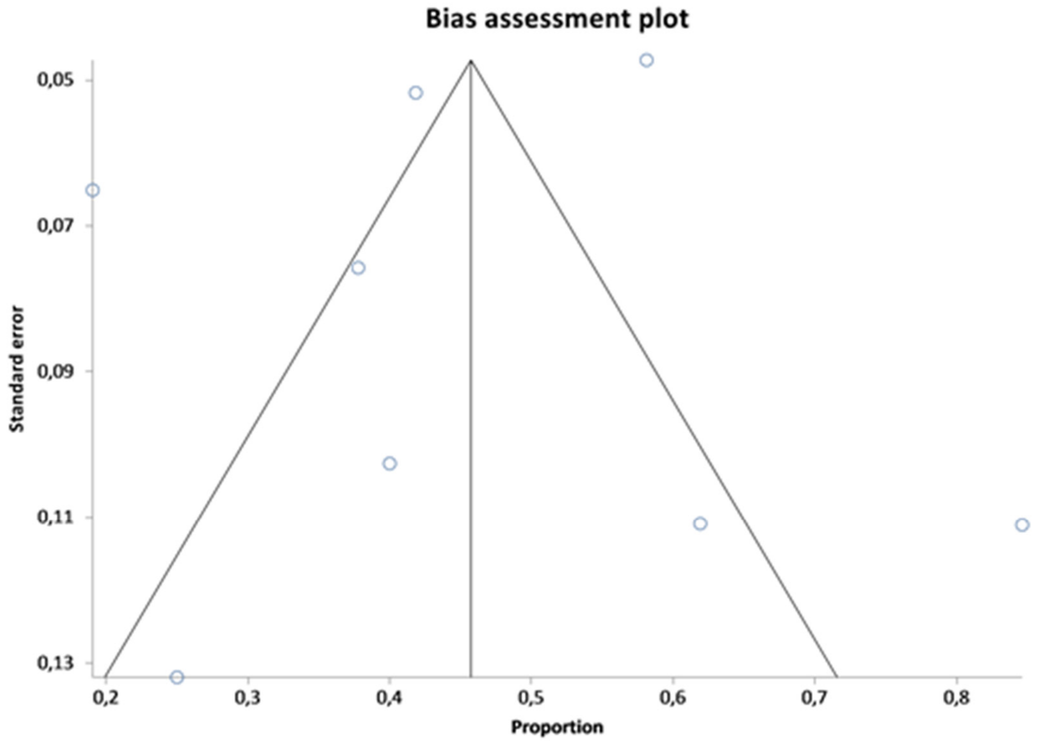

<u>Bias indicators</u>

Egger: bias = 0.1 (95% CI = -6.5 to 6.8) P = 1.0

Figure S5: Funnel Plot for pooled proportion in AIN3

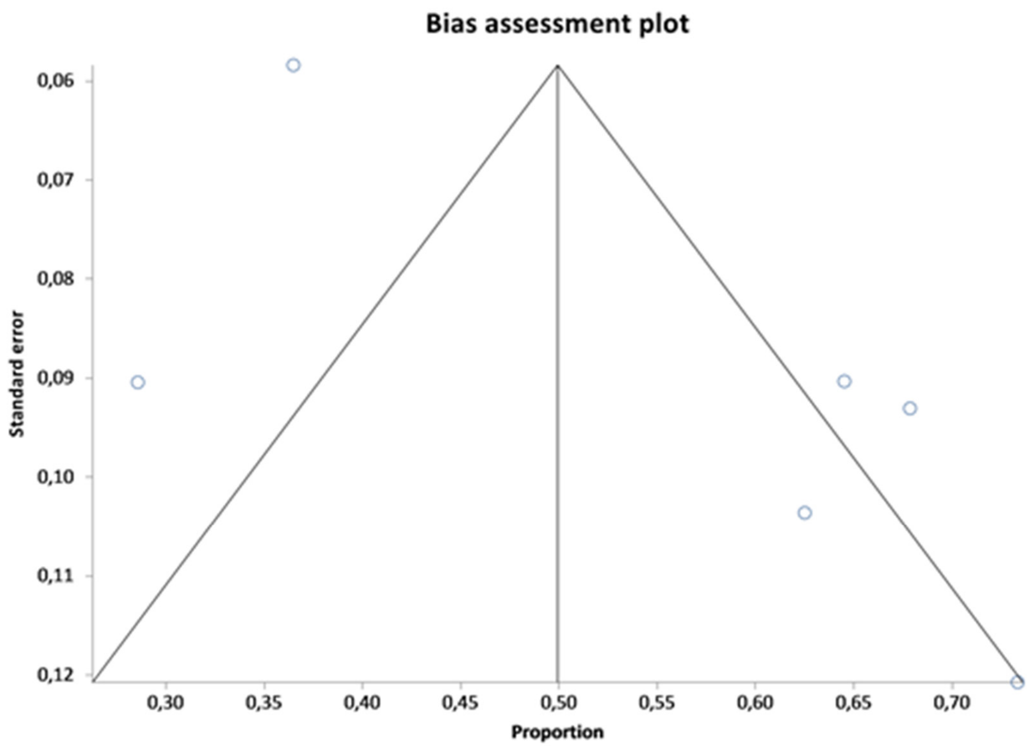

<u>Bias indicators</u>

Egger: bias = 5.8 (95% CI = -2.2-13.7) P = 0.1

Figure S6: Funnel Plot for pooled proportion in SCC

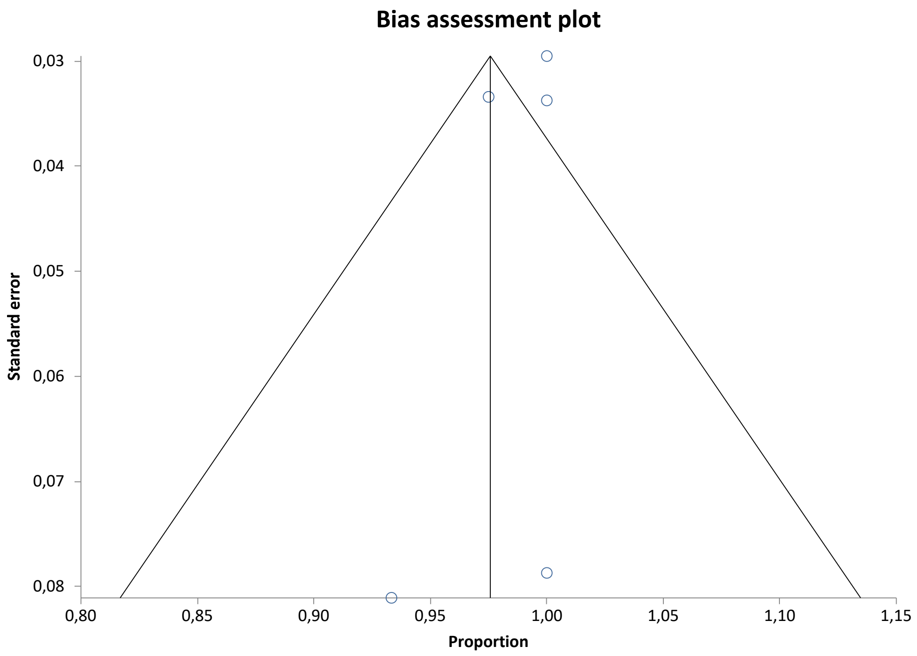

<u>Bias indicators</u>

Egger: bias = -0.6 (95% CI = -2.5-1,3), P = 0.4
